# Using a Single Daytime Performance Test to Identify Most Individuals at High-Risk for Performance Impairment during Extended Wake

**DOI:** 10.1038/s41598-019-52930-y

**Published:** 2019-11-13

**Authors:** Melissa A. St. Hilaire, Bruce S. Kristal, Shadab A. Rahman, Jason P. Sullivan, John Quackenbush, Jeanne F. Duffy, Laura K. Barger, Joshua J. Gooley, Charles A. Czeisler, Steven W. Lockley

**Affiliations:** 1 Division of Sleep and Circadian Disorders, Brigham & Women’s Hospital, 221 Longwood Avenue, Boston, MA 02115 USA; 2 Division of Sleep Medicine, Harvard Medical School, 221 Longwood Avenue, Boston, MA 02115 USA; 3 Biostatistics and Computational Biology, Dana-Farber Cancer Institute, 450 Brookline Avenue, Boston, MA 02215 USA; 4 Programme in Neuroscience and Behavioural Disorders, Duke-National University of Singapore Medical School, 8 College Road, Singapore, 169857 Singapore

**Keywords:** Neurophysiology, Predictive markers

## Abstract

We explored the predictive value of a neurobehavioral performance assessment under rested baseline conditions (evaluated at 8 hours awake following 8 hours of sleep) on neurobehavioral response to moderate sleep loss (evaluated at 20 hours awake two days later) in 151 healthy young participants (18–30 years). We defined each participant’s response-to-sleep-loss phenotype based on the number of attentional failures on a 10-min visual psychomotor vigilance task taken at 20 hours awake (resilient: less than 6 attentional failures, n = 26 participants; non-resilient: 6 or more attentional failures, n = 125 participants). We observed that 97% of rested participants with 2 or more attentional failures (n = 73 of 151) and 100% of rested participants with 3 or more attentional failures (n = 57 of 151) were non-resilient after moderate sleep loss. Our approach can accurately identify a significant proportion of individuals who are at high risk for neurobehavioral performance impairment from staying up late with a single neurobehavioral performance assessment conducted during rested conditions. Additional methods are needed to predict the future performance of individuals who are not identified as high risk during baseline.

## Introduction

Individuals often obtain insufficient sleep due to social^1,2^, environmental^3,4^, and occupational^5–12^ pressures. In the occupational setting, extended work shifts and reduced sleep duration are associated with increased errors, accidents, and lapses in attention^9,13–16^, resulting in economic, societal, and personal costs and consequences. Laboratory-based studies have demonstrated significant inter-individual variability in the response to sleep loss due to extended wake, such that some individuals are able to maintain high levels of neurobehavioral performance on tasks of sustained attention, even when they subjectively report sleepiness^17,18^, whereas other individuals appear to be particularly vulnerable to sleep loss. Identifying individuals who might be especially vulnerable to the neurobehavioral consequences of sleep loss could be useful to prevent errors, accidents, and injuries during and following extended work shifts via countermeasures such as shift reassignment, enhanced supervision, or use of interventions such as light, naps, exercise, and/or caffeine.

Despite evidence that response to sleep loss appears to be trait-like, and therefore consistent within an individual across multiple exposures to sleep deprivation^17,19^, previous attempts at prospectively predicting individual responses to sleep loss have been only moderately successful. One recent model was able to classify individuals studied under highly controlled inpatient conditions as resilient or vulnerable at ~24 hours of sleep deprivation using measures derived from a baseline PVT with an overall accuracy of 77–82%^20^. Similarly, a previous model developed by our group^21^ was able to classify individuals studied under controlled laboratory conditions as resilient, intermediate, or vulnerable to sleep loss with an overall accuracy of 73–75%, but was less accurate (27–56%) in classifying the response to sleep loss in individuals under operational conditions. Other models that have been developed to predict individual response to sleep loss require real-time monitoring over several hours or days that include sleep deprivation^22–24^, which is not practical as a screening tool for prospectively identifying individuals at high risk. The advantage of previous approaches^20,21^, therefore, is that predictions about response to sleep loss can be generated from a single baseline measurement without the need to monitor participants over time or expose them to additional sleep deprivation.

Several recent polls have reported that an estimated 5%-14% of adults say they obtain fewer than 5 hours of sleep per night^25–27^, indicating that a significant proportion of the population is awake for more than 19 hours of each 24 hour day. Furthermore, rates of insufficient sleep have been found to be even higher in individuals in safety-sensitive occupations^28^. The goal of the present analysis was to develop a metric that can predict an individual’s neurobehavioral response to moderate sleep loss (~20 hours awake) from neurobehavioral performance data collected two days earlier during a single rested baseline testing session (after ~8 hours awake following an 8-hour sleep opportunity). The 20-hour time point is relevant as it is analogous to driving home after a long shift, especially when time to prepare for work and commute times are incorporated. We have found that attentional failures on a single neurobehavioral performance assessment when rested are sufficient to identify a high-risk subset of healthy young individuals who are non-resilient after 20 hours awake in this sample of 151 young participants.

## Results

### Response to future sleep loss can be identified by number of attentional failures on a daytime baseline PVT

**Figure 1:**
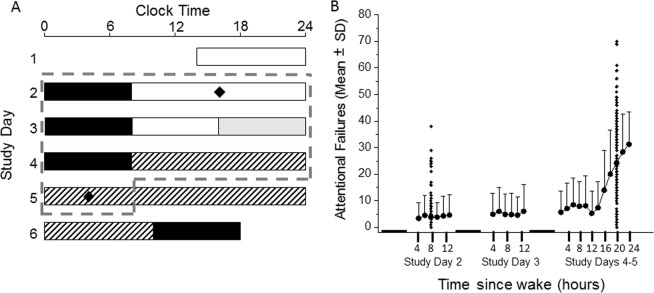
Experimental protocol and average number of attentional failures across all participants. (**A**) Participants completed a 9–10 day inpatient protocol, the first 5 days of which are shown here and were included in the present analysis. Participants entered the laboratory on study day 1 and were scheduled to three consecutive sleep opportunities (black bars, in 0 lux) at their habitual time, as determined by the 7 days prior to admission. Light levels during wake on study days 1 and 2 were ~90 lux (white bars). Halfway through study day 3, light levels were dimmed to ~1 lux (gray bars), a level at which they remained for the remainder of the study during wake. On study days 4–6, participants completed an ~30–50-hour constant routine (CR) procedure (hatched bars, 50 hours is shown) in which they were kept awake in a semi-recumbent posture in dim light and fed equicaloric snacks every hour. Following this CR procedure, participants were scheduled to an 8-hour sleep episode at a time shifted 10 hours relative to their habitual time before completing the remainder of the study. Neurobehavioral measures were collected frequently (every 30 minutes – 2 hours during wake), of which measures collected on study day 2 at +8 h after waking and study day 5 during CR at +20 h after waking were used in the present analysis. (**B**) The mean (±standard deviation) number of lapses (reaction time >500 ms) on the psychomotor vigilance task (PVT) across n = 151 subjects for study days 2, 3, and 4, and up to 24 hours awake during CR on study day 5 (thick gray dashed line in panel A) are shown (filled circles). Individual data points (filled diamonds in panel B) for study day 2 at ~8 h awake and study day 5 at ~20 h awake, which were the focus of analysis in this study, are also plotted. Black bars indicate times of scheduled sleep relative to test times.

Prior definitions of response to sleep loss have been based almost exclusively on performance on the PVT. Thus, we first tested whether incorporating data from other neurobehavioral tests provided additional information about response phenotypes. Our initial dataset included 167 young healthy participants (18–30 years old; 108 males, 59 females) who were studied between 2001 and 2011 in the Intensive Physiological Monitoring unit at Brigham & Women’s Hospital in a 9–10 day inpatient laboratory study of circadian rhythms, as described previously^29–32^ (Fig. 1A). All participants completed the same performance assessment every 2 hours during wake, consisting of the Karolinska Sleepiness Scale (KSS), non-numeric bipolar Visual Analog Scales (VAS), 10-minute auditory psychomotor vigilance task (aPVT), 4-minute addition calculation test (ADD), 1.5-minute digit symbol substitution test (DSST), and 10-minute visual PVT (vPVT).

**Figure 2:**
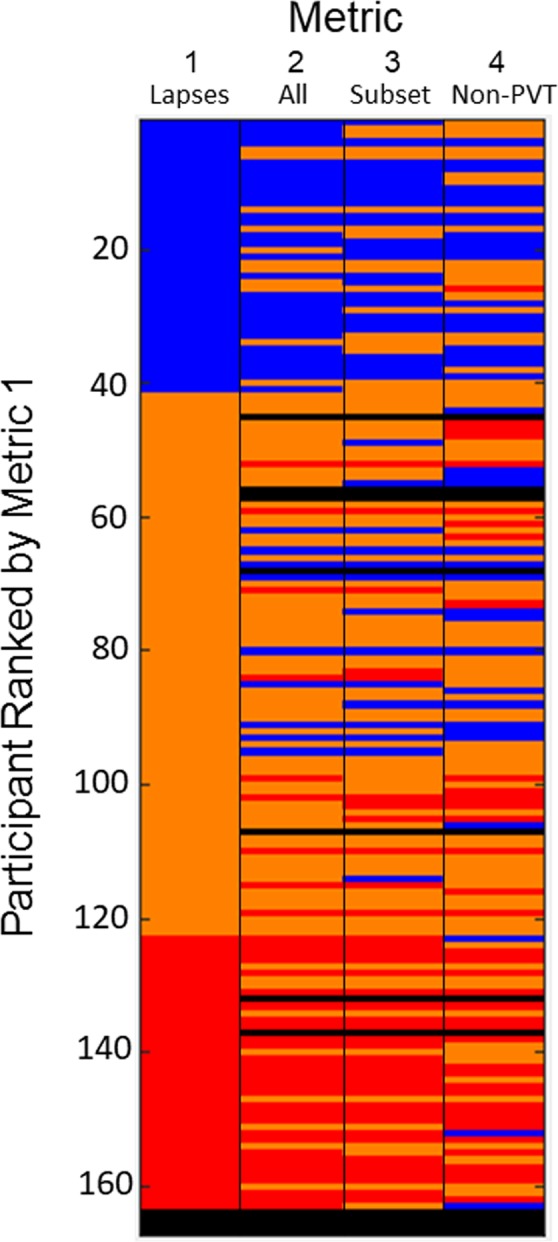
Analysis of response type at ~20 h awake across four metrics. The color map (resilient, blue; intermediate, orange; vulnerable, red; missing data, black) indicates the response phenotype of each participant (n = 167) as determined by the 25^th^ and 75^th^ percentiles of each metric [Metric 1: absolute number of attentional failures (Lapses); Metric 2: first principal component across all 74 variables (All); Metric 3: first principal component across 8 variables (Subset); Metric 4: first principal component across 12 variables (Non-PVT)] at ~20 h awake during constant routine; each row represents one participant and each column represents one metric.

**Figure 3:**
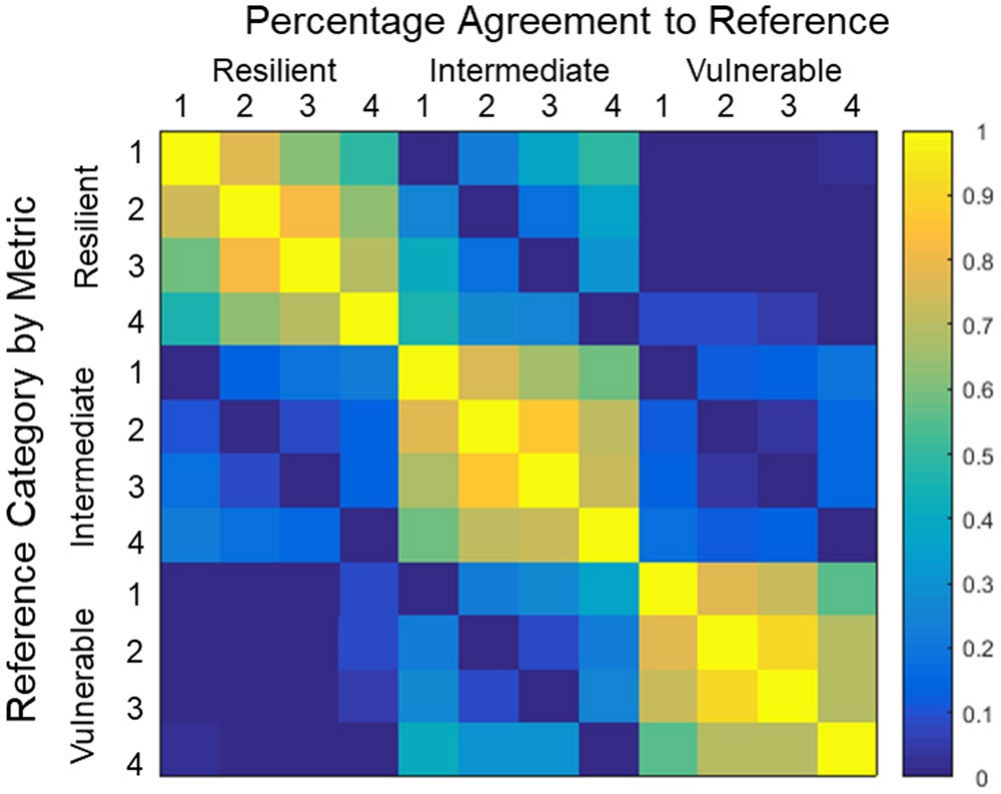
Response type agreement across metrics. The color map indicates the percentage agreement between each of the metrics [Metric 1: absolute number of attentional failures (Lapses); Metric 2: first principal component across all 74 variables (All); Metric 3: first principal component across 8 variables (Subset); Metric 4: first principal component across 12 variables (Non-PVT)] for each response phenotype (resilient, intermediate, vulnerable). Yellow indicates 100% agreement; blue indicates 0% agreement.

We explored four metrics (Table S1) for defining the response to sleep loss phenotype: attentional failures on the vPVT only (Metric 1) (Fig. 1B); a metric derived from principal component analysis of 74 variables from all 6 neurobehavioral tests, including vPVT (Metric 2); a metric derived from principal component analysis of 8 representative variables (Metric 3; mean RT on the vPVT, number of attentional failures on the vPVT, KSS score, VAS Alert score, number attempted on the ADD, number attempted on the DSST, mean RT on the aPVT, and number of lapses on the aPVT); and a metric derived from principal component analysis of 12 variables on all neurobehavioral tests excluding the vPVT and aPVT (Metric 4). Percentiles were computed for each metric derived from data at 20 hours awake. Participants were ranked by percentile, and these percentiles were used as threshold cutoffs for each response type. In our initial analysis, the 25^th^ and 75^th^ percentiles were used to define resilient and vulnerable response phenotypes, respectively; all individuals between the 25^th^ and 75^th^ percentiles were considered intermediate response phenotypes. The heat map in Fig. 2 shows the response phenotype of each participant based on each metric. A comparison across metrics shows that the response phenotypes defined by Metrics 2 and 3 are more similar to those defined by Metric 1 (Fig. 3**)**. These results indicate that similar information is obtained from the three metrics that include data from the PVT (Metrics 1–3) compared with data from other neurobehavioral tasks (Metric 4), and more specifically, that PVT attentional failures alone are sufficient to categorize response phenotype.

### Number of attentional failures on the PVT when rested are sufficient to identify a sub-population of individuals with the non-resilient phenotype two days later

**Figure 4:**
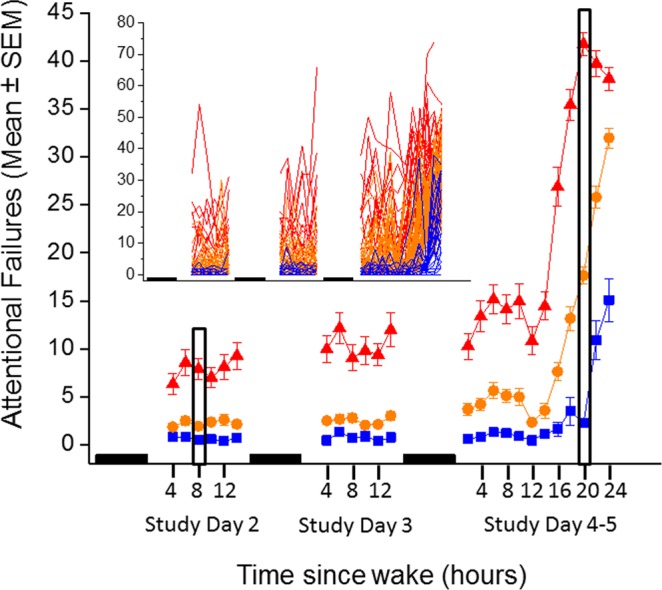
Number of attentional failures on the visual psychomotor vigilance task (PVT) by response phenotype. Participants (n = 151 who had data at both ~8 h awake and ~20 h awake) were stratified by response phenotype (resilient, intermediate, and vulnerable in blue, orange, and red, respectively) as determined at ~20 h awake and averaged within each group for each test battery. Data are plotted as the mean and standard error of the mean (SEM). The inset shows the individual data. The filled black boxes indicate relative sleep opportunities and the black outlined areas indicate the time windows that were used for the current analysis.

Based on the distribution of attentional failures on the vPVT (Metric 1) at ~20 h awake, we defined the following response phenotypes: a participant with fewer than 6 attentional failures (20^th^ percentile) was considered resilient, a participant with greater than 30 attentional failures (60^th^ percentile) was considered vulnerable, and a participant with between 6 and 30 attentional failures was considered intermediate. The individual and grouped average data for all subjects stratified by these three groups based on these definitions are shown in Fig. 4.

**Figure 5:**
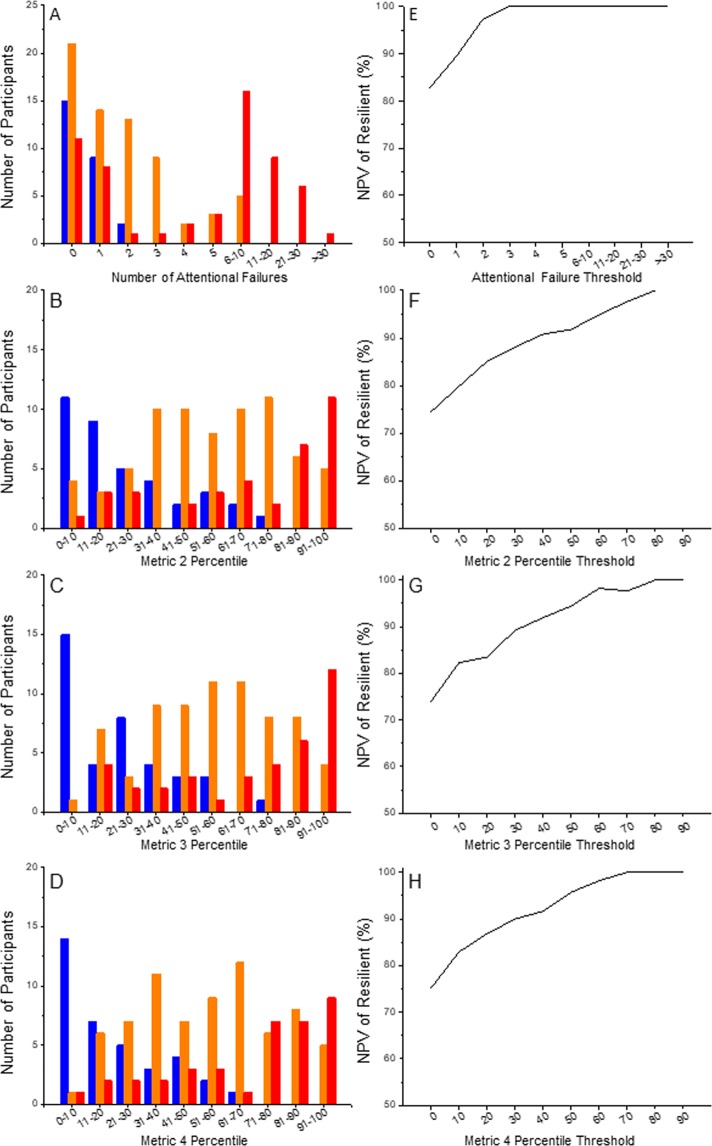
Distribution of response to sleep loss phenotypes at baseline. Histograms of the number of attentional failures (**A**) and the percentile ranks of Metrics 2, 3, and 4 (**B-D**, respectively) observed at ~8 h awake during baseline (study day 2, Fig. 1) are stratified by response phenotype at ~20 h awake (resilient, blue; intermediate, orange; vulnerable, red). The corresponding negative predictive value (NPV) for non-resilient response phenotypes (i.e., intermediate or vulnerable) is plotted for each metric in panels (**E–H**).

We next extended these analyses by examining the distribution of response phenotypes at ~20 h awake as a function of the value of each metric at ~4, ~6, and ~8 h awake following an 8-hour sleep opportunity during baseline 2 days prior; we focus here on the data from ~8 h awake as it was found to be the most robust in partitioning non-resilient from resilient phenotypes (Fig. 5**)**. Figure 5A–D show the distribution of values at ~8 h awake for each metric, and Fig. 5E–H show the negative predictive value (NPV) curves for each metric. Based on these analyses, application of Metric 1 at 8 hours awake (i.e., number of baseline attentional failures) enables a single split that excludes (i.e., labels as non-resilient) a significant proportion of individuals who are non-resilient at 20 hours awake two days later. At ~8 hours awake two days prior to the constant routine, 73 participants (48%) in our sample had 2 or more attentional failures on the vPVT, of whom 32 (44%) were found to be intermediate, 39 (53%) were found to be vulnerable, and only 2 (3%) were found to be resilient at ~20 h awake two days later after two more nights of 8 hour sleep. All participants with 3 or more attentional failures on the vPVT at ~8 h awake (n = 57) were found to be non-resilient (n = 19, 33% intermediate; n = 38, 67% vulnerable) two days later at ~20 h awake. Thus, the negative predictive value for a resilient response type (i.e., prospectively identifying individuals who are non-resilient) is 97% for 2 or more attentional failures and 100% for 3 or more attentional failures on the vPVT. Specifically, the number of baseline attentional failures identifies more than half (57%) of all individuals who will be non-resilient to moderate sleep loss (~20 h awake) two days later.

In comparison, application of Metrics 2, 3, and 4 also enables single splits, identifying sub-populations of 43, 56, and 57 individuals, respectively, at ~8 h awake when rested who are non-resilient at ~20 h awake. Thus, Metrics 2, 3, and 4 are each less powerful at identifying non-resilient individuals than Metric 1. Furthermore, Metrics 2, 3, and 4 are more complex to determine and more sensitive to population specificity (due to the use of principal component analysis) than Metric 1, each has more missing data than Metric 1 (analysis of Metrics 2, 3, and 4 included only 145, 146, and 145 individuals, respectively), and each erroneously misidentifies one resilient individual.

Exclusion of subjects with 2 or more attentional failures when rested yields an enriched subset of high-level performers with 0 or 1 attentional failure at ~8 h awake, with a more equitable distribution of response to sleep loss phenotypes: n = 24 (31%) resilient, n = 35 (45%) intermediate, and n = 19 (24%) vulnerable. For these individuals (n = 78), attentional failures on the vPVT when rested are alone insufficient to discriminate response to sleep loss phenotype; additional methods would be needed to accurately predict the phenotypes of these high-level performers to differentiate non-resilient from resilient individuals in this group.

### Sensitivity of Metric 1 at other time points

**Figure 6:**
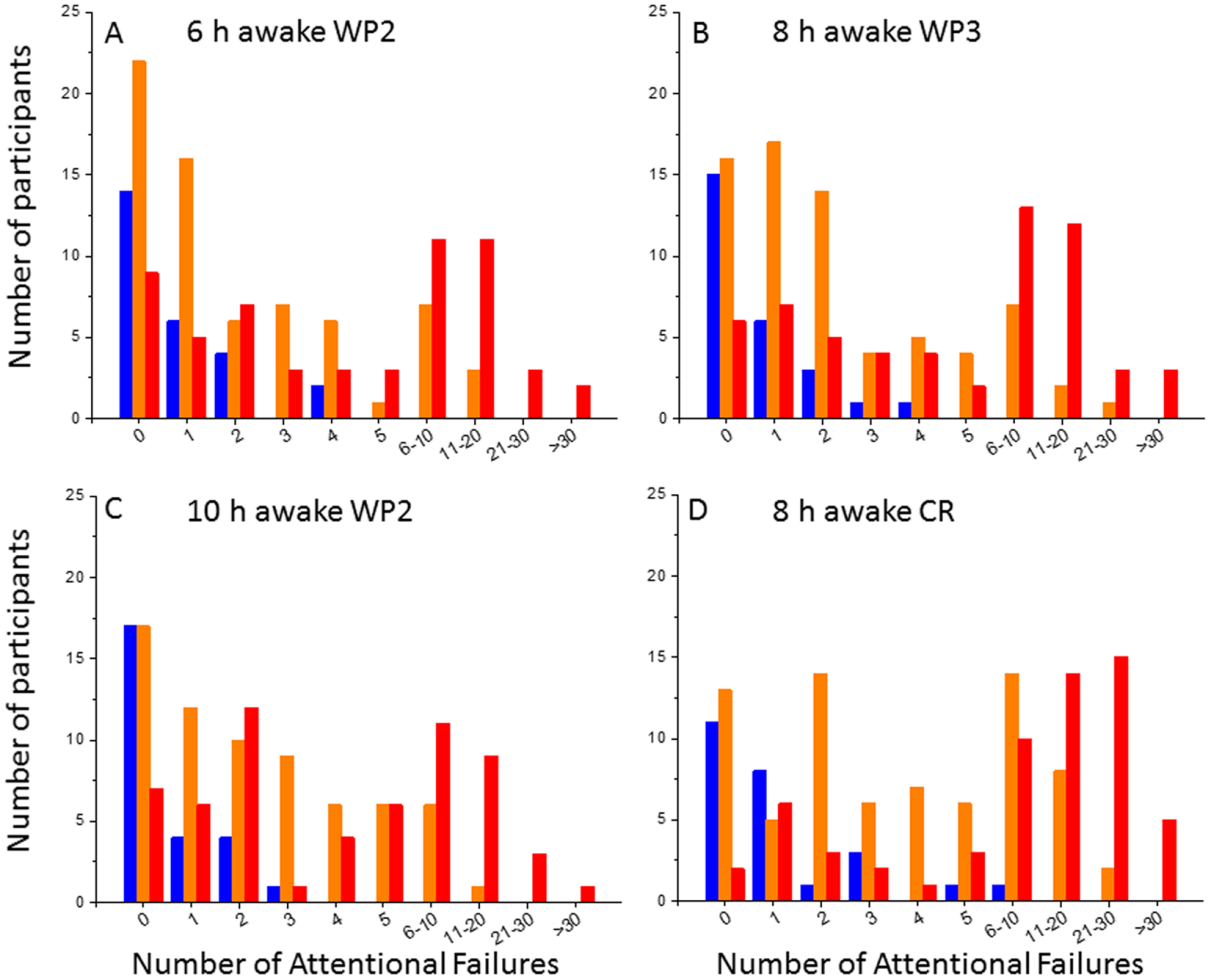
Distribution of response to sleep loss phenotypes under different baseline test sessions. Histograms of the number of attentional failures observed at ~6 h awake on study day/wake period 2 (WP2), ~10 h awake on WP2, ~8 h awake on study day/wake period 3 (WP3), and ~8 h awake on constant routine (CR). Attentional failures are stratified by response phenotype at ~20 h awake (resilient, blue; intermediate, orange; vulnerable, red). These observations confirm the finding from Fig. 5A (~8 h awake WP2) that a single partition at <2 versus 2 or more attentional failures can robustly identify a subset of participants who exhibit intermediate or vulnerable phenotypes at ~20 h awake across several time points.

To determine whether this finding is robust at other time points, we also tested the single split at <2 versus 2 or more attentional failures at 6 and 10 hours awake on study day 2 (i.e., wake period 2 [WP2]) as well as 8 hours awake on study day 3 (WP3) and 8 hours awake on the CR day. The findings, which are reported in Fig. 6, indicate that this single split is robust, and can identify individuals who will be non-resilient at 20 hours awake with a consistently high NPV at multiple time points, including a time associated with a “post-lunch dip” in performance (6 hours awake) and close to the wake-maintenance zone (10 hours awake). We did not test earlier than 6 hours awake here due to potential confounding of sleep inertia and we did not test later than 10 hours awake due to potential confounding of the wake-maintenance zone. As expected, the number of individuals who are identified correctly as poor performers from a single PVT session increases the closer the baseline test occurs to the time point of interest. Overall, applying the single split of <2 versus 2 or more attentional failures on any of these single PVT sessions can identify anywhere from 47–68% (mean ± SD 55% ± 8%) of non-resilient individuals while minimizing the number of resilient individuals incorrectly predicted as non-resilient (3% ± 1% of all participants at each time point tested).

Combining information from multiple PVT sessions further improves our ability to identify individuals who are non-resilient to sleep loss at ~20 hours awake. For example, 50 individuals are identified as poor performers (i.e., 2 or more attentional failures) on all three PVT sessions conducted at 6, 8, and 10 hours awake on WP2, which represents approximately one-third of participants in our analysis (n = 146 with valid results on all 3 baseline PVT sessions and at ~20 h awake on CR session), and all 50 of these individuals are defined as non-resilient at ~20 h awake two days later (i.e., 100% NPV). If we examine individuals who exhibit 2 or more attentional failures on at least one PVT session across WP2 (i.e., at 6, 8, or 10 hours awake, n = 157 with valid test results for at least one time point) or across days (i.e., at 8 h awake on WP2, WP3, and/or CR, n = 163 with valid test results for at least one time point), then we are able to identify an even higher proportion of individuals who are non-resilient to sleep loss (Table 1), although this approach also misclassifies more individuals as non-resilient who are resilient at ~20 h awake. These findings suggest, therefore, that application of this approach at different time points requires the user to balance the benefit of correctly removing more non-resilient individuals against the cost of incorrectly discarding resilient individuals from the population.

**Table 1:** Prediction of resilient vs. non-resilient individuals at ~20 hours awake based on multiple PVT sessions.

Predictions based on 2 or more attentional failures on at least one PVT session during WP2*		
	Predicted resilient	Predicted non-resilient
Actual resilient	17	9
Actual non-resilient	30	101
Predictions based on 2 or more lapses on at least one PVT session at 8 hours awake**		
	**Predicted resilient**	**Predicted non-resilient**
Actual resilient	17	9
Actual non-resilient	17	120

*PVT sessions conducted at 6, 8, and 10 h awake on study day/wake period 2 (WP2).

**PVT sessions conducted at 8 h awake on WP2, WP3, and constant routine (CR).

### Validation of Metric 1 on an independent data set

**Figure 7:**
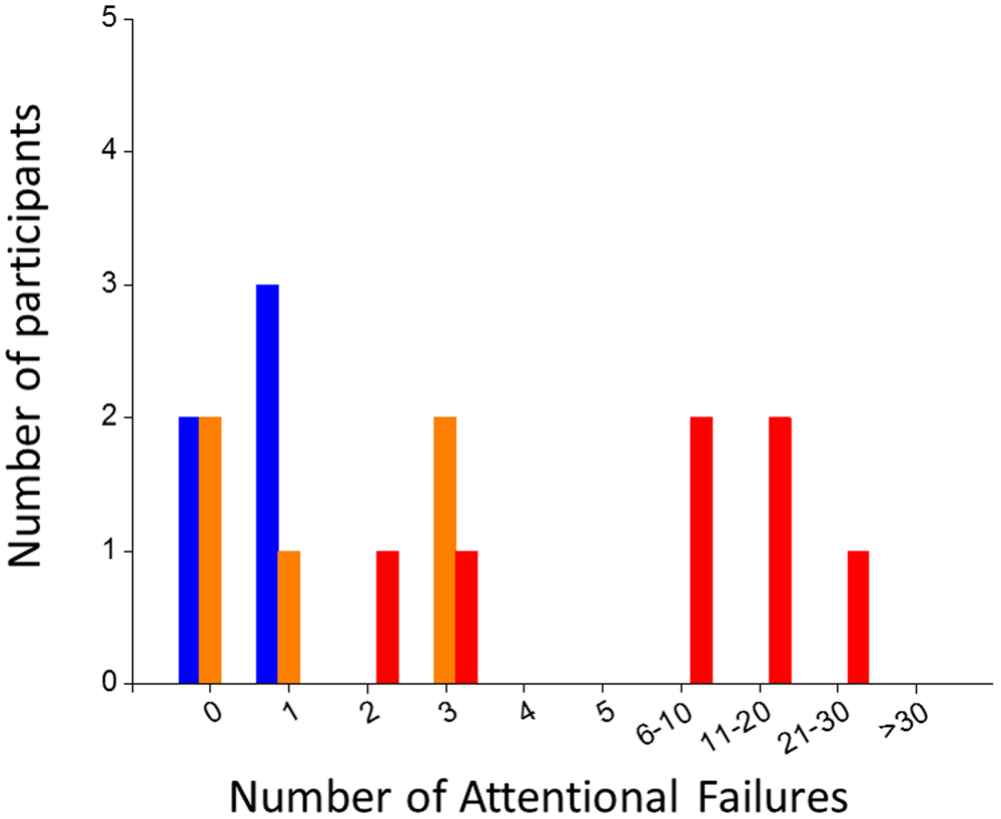
Validation of the use of Metric 1 on an independent data set. Histogram of the number of attentional failures observed at 8 h awake during baseline in n = 17 participants who completed an experimental protocol similar to Fig. 1. Attentional failures are stratified by response phenotype at 20 h awake (resilient, blue; intermediate, orange; vulnerable, red). These observations indicate that a single partition at <2 versus 2 or more attentional failures can robustly identify a subset of participants who exhibit intermediate or vulnerable phenotypes at 20 h on an independent data set.

To validate the use of Metric 1 to differentiate between high-level and poor performances when rested, we tested the single split at <2 versus 2 or more attentional failures at ~8 h awake on WP2 on an independent data set of n = 17 participants^33^ who were studied under the same laboratory conditions as our original data set of 167 participants. All 9 participants with 2 or more attentional failures at ~8 h awake on WP2 were intermediate (n = 2) or vulnerable (n = 7) at ~20 h awake two days later (Fig. 7). Of the 8 subjects who had 0 or 1 attentional failure at ~8 h awake on WP2, 5 were resilient and 3 were intermediate at ~20 h awake two days later. These findings confirm the robustness of our approach on an independent data set.

### Application of Metric 1 on prospective study participants

**Figure 8:**
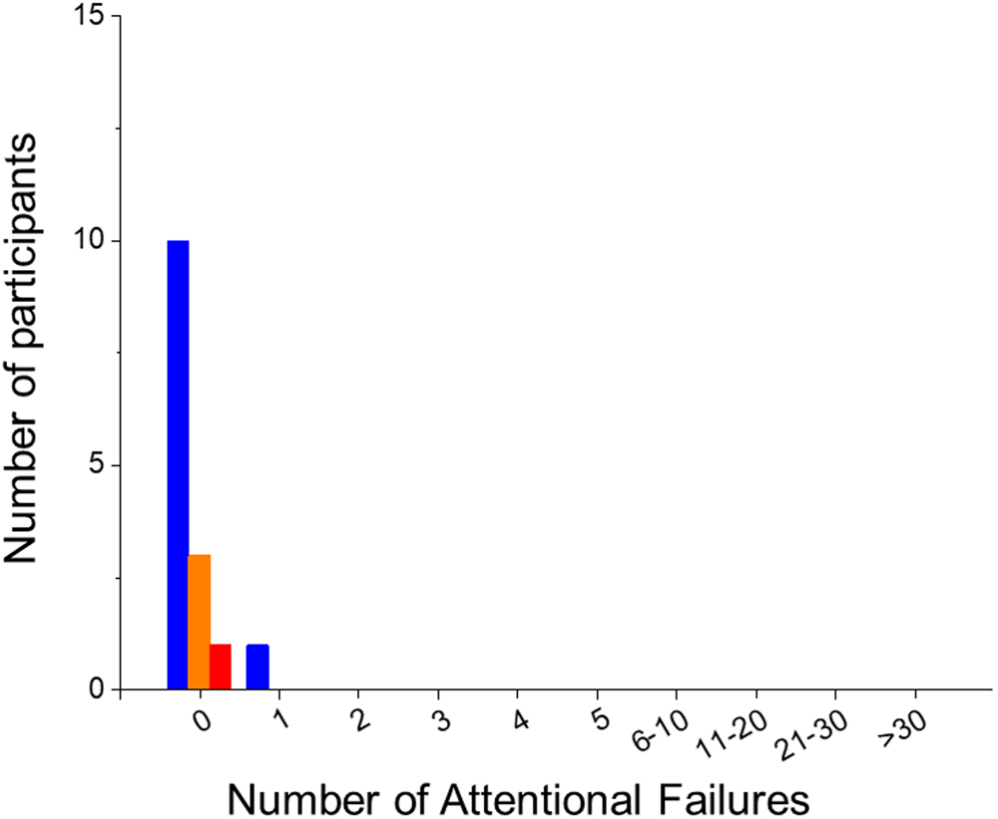
Application of Metric 1 to prospective study participants. Histogram of the number of attentional failures observed at ~8 h awake during baseline in n = 15 participants who completed an experimental protocol similar to Fig. 1A. Attentional failures are stratified by response phenotype at ~20 h awake (resilient, blue; intermediate, orange; vulnerable, red). Participants in this study completed a series of outpatient screening test batteries at 4, 6, and 8 h awake; only participants who exhibited 0 or 1 attentional failure on at least two of the three test sessions were allowed to continue onto the inpatient portion of the study.

We prospectively tested the use of Metric 1 to screen participants for an inpatient study of acute sleep deprivation. Prospective healthy volunteers (males and females, ages 26–55 years old) were asked to maintain an 8-hour time-in-bed schedule for 1 week, and then visited our outpatient facility to complete three test batteries (identical to the test battery administered to participants included in the present analysis) at 4, 6, and 8 h after their habitual wake time. Participants who exhibited 2 or more attentional failures on at least two of these three test batteries were excluded from subsequent participation in a 6-day inpatient study, which followed the same schedule as presented in Fig. 1A. Of 34 prospective participants who completed this outpatient screening test, 26 met our test battery criteria (0 or 1 attentional failure on at least two tests), and 15 completed the study. All participants who completed the inpatient study (n = 15) were confirmed to be high-level performers at ~8 h awake during WP2 (Fig. 8). Furthermore, the majority of these participants (n = 11, ~73%) were classified as resilient at ~20 h awake. When we apply these same criteria to our original data set of 167 participants, we note that 79 of 151 participants who had valid PVT data at 4, 6, and 8 h awake on WP2 and at 20 h awake on CR met these criteria. Of these 79 participants, 23 were resilient. Of the 72 participants who did not meet these criteria, only 3 were resilient and would have been excluded from the inpatient study. These results suggest that Metric 1 can be used prospectively to target high-level performers on a slightly older population than the population on which the metric was developed (i.e., 26 to 55 years old versus 18 to 30 years old).

## Discussion

We have defined two levels of performance ability: (1) high *versus* poor performance when rested and (2) level of performance impairment (resilient, intermediate, or vulnerable phenotype) in response to a single bout of moderate acute sleep loss. Our analysis of neurobehavioral performance at ~20 h awake showed that the number of attentional failures on a single 10-minute vPVT test was sufficient to define a response to sleep loss phenotype in all individuals in a healthy young population, as additional neurobehavioral variables did not significantly impact phenotype classification. Thus, attentional failures on the vPVT were the dominant feature for establishing a simple partition below which individuals almost uniformly exhibited poor response to sleep loss. Next, by examining the distribution of performance at ~8 h awake on a baseline day, we determined that all rested participants with 3 or more attentional failures and all but 2 rested participants with 2 or more attentional failures on the vPVT exhibited a non-resilient phenotype after 20 h awake two days later, allowing us to split our population into two subtypes when rested: high-level and poor performers. Poor performers at baseline initially exhibit apparent non-sleep-loss-related impairment on the vPVT, but continue to exhibit declines in performance under sleep deprivation. In contrast, high-level performers exhibit optimal performance when rested. More than two-thirds of these initially high-level performers proved to be non-resilient to sleep loss, whereas nearly a third (31%) continued to exhibit few attentional failures through ~20 h awake. This partition also was robust at identifying future high-level versus poor performers across multiple time points, on an independent data set, and during outpatient screening for an inpatient study.

The ability to identify a subset of non-resilient individuals from a single 10-minute baseline vPVT test provides an important screening tool for identifying a significant proportion of poor-performing individuals who are at greatest risk of suffering additional potentially deleterious performance consequences from sleep loss. Identification of these poor-performing individuals, who represented approximately 50% of our population, can facilitate the implementation of prophylactic interventions to mitigate the consequences of insufficient sleep, including scheduling these individuals to shorter work shifts, providing more break/nap opportunities, administering light exposure therapy timed optimally to improve performance during extended work shifts, and/or providing transportation home to these individuals following an extended shift. One caveat is that we have only included in our analysis individuals who had 8-hour sleep opportunities just prior to the baseline assessments in addition to at least two weeks of 8 hours time-in-bed per night immediately prior to entering the study. These findings may therefore not extend to individuals who routinely obtain less than 8 hours of time-in-bed per night (i.e., those who are chronically sleep deprived) or to those who routinely spend more time in bed (i.e., those for whom 8 hours time-in-bed is not sufficient). In addition, the underlying cause for poor performance on the PVT under our well-rested condition across ~50% of individuals is unknown. The PVT is a monotonous task, and motivation may have been an issue^34^. Another possibility is that a sleep history of 8 hours time-in-bed did not provide sufficient sleep satiation in these individuals; at least one study has shown that a sleep history of 10 hours time-in-bed yields fewer attentional failures on a baseline PVT than a sleep history of 8 hours time-in-bed^35^.

As in essentially any non-trivial predictive modeling problem, there is an inherent trade-off between modeling power and predicted reliability and robustness of the resulting model. The conservative modeling approach taken here has both advantages and disadvantages. The focus on the number of attentional failures on the vPVT as the single critical outcome is somewhat *biologically* disadvantageous in that it minimizes the known inter-individual differences in which functional measures decay with increased sleep restriction. Moreover, the correlation between the number of attentional failures on the vPVT and more operationally relevant tasks, such as driving or more complex work, remains unclear despite widespread use of the vPVT in sleep research. Conversely, the focus on the number of attentional failures on the vPVT as the sole outcome measure is advantageous in that it builds on the general consensus that vPVT is a fairly sensitive measure of response to sleep loss. The results we obtained using number of attentional failures on the vPVT were highly consistent with two other metrics that included, but did not mathematically emphasize, multiple outcome variables from the vPVT (i.e., Metrics 2 and 3), and were moderately consistent with another metric that included outcome variables from multiple non-PVT tasks (i.e., Metric 4). Although the specific partition of attentional failures that we have identified to differentiate resilient (0–5 attentional failures) from non-resilient (greater than 5 attentional failures) performance might be expected to vary in different populations, e.g., drug-treated, elderly, high-performing professionals, shift-workers etc., the data predict that this single partition model (i.e., the number of attentional failures on the vPVT itself) will have significant reliability and predictability in most or all datasets. Thus, this study provides a dependable “floor” as to the ability of this approach to discern a significant proportion of individuals who are at highest risk of impairment in response to sleep loss (and, reciprocally, to enrich for those who are at lower risk).

Our highly homogenous population is both a strength and limitation of the approach developed in this study. All participants in the current analyses were young and healthy, studied under highly controlled inpatient laboratory conditions after two or more weeks of maintaining a consistent 8-hour time-in-bed each night at home, and restricted from the use of substances that are known to affect neurobehavioral performance, such as caffeine. We expect that each participant was performing close to their own best level in the baseline condition based on the participants’ sleep-wake history, which included selection of individuals with a self-reported history of 7–9 hours of sleep per night, maintenance of an 8-hour time-in-bed schedule for at least two weeks at home immediately prior to the laboratory admission, and 8 hours time-in-bed in the laboratory the previous night. Although such rested conditions are not likely to be encountered in real-world settings, most individuals have access to other performance enhancers, such as caffeine and bright light, which were restricted in our study. In addition, the constant routine conditions, including a constant semi-recumbent posture in dim light, likely induced a greater level of sleepiness at ~20 h awake than would be expected in an operational setting; thus, we may have overestimated the number of individuals who would be categorized as intermediate or vulnerable at ~20 h awake. Further work is needed to determine the robustness of our approach among individuals studied under more realistic conditions, including in the presence of caffeine and other stimulants that may reduce the overall number of attentional failures as well as under conditions that may further increase the number of attentional failures, such as chronic sleep deficiency, stress, illness, distractions, and other factors. Nevertheless, the robust finding that rested individuals who showed 3 or more attentional failures exhibited further impairment under moderate sleep loss is therefore likely to be highly reproducible under a range of real-world settings.

At present, individuals who are identified by our metric as high-level performers when rested cannot be differentiated as resilient or non-resilient when challenged two days later with moderate acute sleep loss from a single baseline neurobehavioral performance assessment, and thus will require an additional diagnostic test to determine *a priori* whether they will exhibit significant neurobehavioral impairment beyond 20 hours of wakefulness. This approach is not different from clinical screening and diagnosis procedures for many diseases. For example, the majority of pregnant women are screened for gestational diabetes via a 1-hour oral glucose tolerance test. Pregnant women whose glucose levels exceed a pre-defined threshold (typically 140 mg/dL) 1 hour after consuming a glucose drink must then complete a 3-hour version of the oral glucose test. After this second test, only those women whose glucose levels exceed pre-defined thresholds at 1, 2 and/or 3 hours after the glucose test are diagnosed and treated for gestational diabetes. Our metric therefore provides the initial screening test to separate those who are definitely non-resilient from those who may or may not be non-resilient. Our inability to further differentiate high-level performers at baseline as non-resilient or resilient highlights the need for a further diagnostic test, e.g., a biomarker of response to sleep loss independent of neurobehavioral function that can be derived from other physiological measures. Several such biomarkers have been proposed, but none have been tested within the type of modeling framework described here. For example, Chua *et al*.^18^ reported that participants who were differentiated into resilient and vulnerable subtypes during sleep deprivation on the basis of PVT measures also exhibited significant differences in percentage of eye closure and blink rate, heart rate, and EEG spectral power in the theta frequency band. Functional connectivity as measured by resting-state functional magnetic resonance imaging is also different between resilient and vulnerable individuals^36^. Furthermore, individuals with the long homozygous polymorphism of the PERIOD3 clock gene (Per3^5/5^) have also been reported to show greater detrimental effects of sleep loss on the PVT compared with individuals who are homozygous for the short homozygous (PER3^4/4^) allele^37^, which lends support to the trait-like characteristic of response to sleep loss. In addition, recent studies have demonstrated sleep-dependent changes in the human plasma metabolome^38–41^, which indicates the possibility that a biomarker in blood may eventually be discovered that can predict an individual’s response to sleep loss phenotype without the need to subject them to sleep deprivation. Further research is needed to determine whether such measures collected after an adequate sleep duration (e.g., 8 hours) already reflect differences between resilient and vulnerable individuals as a way to *a priori* identify response to sleep loss.

The screening tool developed in this study could have a significant impact on the health and safety of not only individuals in safety-sensitive occupations, who are either required to or decide to stay awake past the recommended 16–17 hours per day^42^, but also on the general public who depend on those individuals to be operating at sufficient levels of performance across and beyond their work shifts. By being able to identify a significant proportion of the population who may disproportionately contribute the majority of errors following moderate extended wakefulness, resources can be focused on minimizing errors in those most vulnerable individuals. Such a tool is therefore likely to significantly enhance current fatigue and sleep management strategies.

## Methods

### Participants and experimental protocol

The studies from which the data for the present analysis originated were conducted in accordance with the Declaration of Helsinki, and were reviewed and approved by the Partners Human Research Committee in accordance with Title 45, U.S. Code of Federal Regulations, Part 46, Protection of Human Subjects. All study participants provided written informed consent prior to the study.

We compiled data from 167 young healthy participants (18–30 years old) who were studied in the Intensive Physiological Monitoring unit at Brigham & Women’s Hospital in a 9–10 day inpatient laboratory study of circadian rhythms^29–32^, as previously described. The screening and selection criteria and the first five days of these studies were identical, and were included in the present analysis. Briefly, participants were eligible for the inpatient study if they had normal sight and were in good physical and mental health as determined by medical history, physical examination, and psychological questionnaires and interview. Participants also had to be free from caffeine, alcohol, nicotine, medications, and illicit drug use as determined by urine toxicology during screening and at the time of the inpatient admission. Participants kept a very regular sleep-wake schedule (16 hours awake, 8 hours time-in-bed) at their habitual times for at least two weeks prior to the inpatient study. Compliance to this schedule in the seven days before admission to the inpatient study was confirmed by continuous actigraphy monitoring (Actiwatch-L; Minimitter, Inc., Bend, OR). During the inpatient stay, participants lived individually in a study room that was free of time cues. During the first three days of the study, participants were scheduled to sleep and wake at the habitual times derived from the seven days prior to laboratory admission. Participants slept in darkness and were exposed to room light (maximum of ~190 lux/48 µW/cm^2^ when measured in the horizontal plane at a height of 187 cm and ~88 lux/23 µW/cm^2^ when measured in the vertical plane at a height of 137 cm) during waking until midway through study day three, after which the light was dimmed to <3 lux (~1.5 lux/0.4 µW/cm^2^ maximum when measured in the horizontal plane and ~0.6 lux/0.1 µW/cm^2^ when measured in the vertical plane). On the following morning, participants awoke to and began a 30–50-hour constant routine (CR) procedure in which they were kept awake in bed in a semi-recumbent posture under dim light (<3 lux), and were fed hourly equicaloric snacks. This procedure aimed to keep the participant’s environment and behavior as constant as possible across the 30–50 hour monitoring episode. Ambient light was provided by ceiling-mounted 4100 K fluorescent lamps (Philips Lighting, Eindhoven, The Netherlands) and transmitted through a UV-stable filter (Lexan, General Electric Plastics, Pittsfield, MA, USA). A summary of the protocol is provided in Fig. 1A.

In these studies, the KSS^43^ and VAS (Alert-Sleepy, Sad-Happy, Excited-Calm)^44^ were administered every 30 minutes during wake starting ~2 hours after wake time. The aPVT^45^ was administered every 60 minutes. A full test battery consisting of the KSS, VAS, 10-minute aPVT, 4-minute ADD^46^, 1.5-minute DSST^47^, and 10-minute vPVT^48^ was administered every 2 hours. Only data from this full test battery were included in the current analysis. In total, 185 variables were computed from each test battery and included in an initial assessment of potential metrics on which to base a definition of response to sleep loss: 81 variables each from the vPVT and aPVT, one variable from the KSS, three variables from the VAS, two variables from the ADD, and 17 variables from the DSST. Many of these variables were collinear (i.e., provided redundant information), and thus excluded from the final analysis. The final dataset, therefore, included 74 variables: 31 each from the vPVT and aPVT, one from the KSS, three from the VAS, two from the ADD, and six from the DSST (Table S1).

### Defining response to sleep loss at ~20 hours awake

In the first stage of our analysis, we developed a definition of response to sleep loss based on neurobehavioral performance at 20 hours awake during CR, reflecting moderate sleep loss. We chose *a priori* to examine four metrics (Table S1) on which to base our definition of response to sleep loss. The first metric was based on the absolute number of attentional failures (reaction times > 500 ms) on the vPVT at 20 hours awake. The second and third metrics were based on principal component analyses across all 74 variables (Metric 2) and across a subset of 8 representative variables from the different neurobehavioral tests [mean RT and number of lapses on the vPVT, KSS score, VAS Alert score, number attempted on the ADD, number attempted on the DSST, and mean RT and number of lapses on the aPVT] (Metric 3). The fourth metric was based on principal component analyses across 12 variables from KSS, VAS, ADD, and DSST only (i.e., excluding vPVT and aPVT variables). For Metrics 2, 3, and 4, principal component analysis (function ‘pca’ in MATLAB, The MathWorks, Natick, MA, USA) was applied to the variables at ~20 h awake across all participants, and the first principal component was extracted for further analysis. We analyzed different percentile cutoffs for defining three phenotypes of response to sleep loss: resilient, intermediate, and vulnerable.

### Predicting response to sleep loss during rested conditions

In the second stage of our analysis, we examined the value of each metric in each participant during rested conditions (evaluated at 8 hours awake following an 8-hour sleep opportunity two days prior to the CR) as a function of their response to sleep loss phenotype defined at 20 hours awake from that same metric. We calculated the negative predictive value of different cutoffs for each metric to determine which cutoff for each metric could best separate resilient from non-resilient phenotypes using a single neurobehavioral performance assessment collected during rested conditions.

## Supplementary information

Supplementary Materials

## Data Availability

The authors will make de-identified data from the current study available upon written request. Execution of a Materials Transfer Agreement is required if the data will be used in research supported by a for-profit company, per Partners Healthcare Institutional Review Board policy.
