# Supplementary material for: Using a Single Daytime Performance Test to Identify Most Individuals at High-Risk for Performance Impairment during Extended Wake
Source: Sci Rep. 2019 Nov 13;9:16681. doi: 10.1038/s41598-019-52930-y (PMC6853981; doi:10.1038/s41598-019-52930-y)
Supplement: Supplementary file 1 — Supplementary Materials [file 41598_2019_52930_MOESM1_ESM.docx]

**Supplementary Materials**

**Title:** **Using a Single Daytime Performance Test to Identify Most Individuals at High-Risk for Performance Impairment during Extended Wake**

**Authors:** Melissa A. St. Hilaire^1,2^*, Bruce S. Kristal^1,2^, Shadab A. Rahman^1,2^, Jason P. Sullivan^1^, John Quackenbush^3^, Jeanne F. Duffy^1,2^, Laura K. Barger^1,2^, Joshua J. Gooley^4^, Charles A. Czeisler^1,2^, and Steven W. Lockley^1,2^

**Author Affiliations:**

^1^Division of Sleep and Circadian Disorders, Brigham & Women’s Hospital, 221 Longwood Avenue, Boston, MA 02115;

^2^Division of Sleep Medicine, Harvard Medical School, 221 Longwood Avenue, Boston, MA 02115;

^3^Biostatistics and Computational Biology, Dana-Farber Cancer Institute, 450 Brookline Avenue, Boston, MA, 02215

^4^Programme in Neuroscience and Behavioural Disorders, Duke-National University of Singapore Medical School, 8 College Road, Singapore 169857

*To whom correspondence should be addressed: [msthilaire@rics.bwh.harvard.edu](mailto:msthilaire@rics.bwh.harvard.edu)

**Table S1.** Neurobehavioral variables included in each metric and 1^st^ principal component loading (when applicable).

| **Variable** | **Description** | **Metric 1** | **Metric 2** | **Metric 3** | **Metric 4** |
| --- | --- | --- | --- | --- | --- |
| VPVT_ANTICIPATIONS | Count of responses recorded prior to visual stimulus presentation |  | 0.01 |  |  |
| VPVT_ALL_MEAN | Average of all reaction times during the vPVT session |  | 0.17 | 0.42 |  |
| VPVT_ALL_MED | Median of all reaction times during the vPVT session |  | 0.16 |  |  |
| VPVT_ALL_STD | Standard deviation of all reaction times during the vPVT session |  | 0.15 |  |  |
| VPVT_SLOW_MEAN | Average of slowest 10% of reaction times during the vPVT session |  | 0.15 |  |  |
| VPVT_SLOW_STD | Standard deviation of slowest 10% of reaction times during the vPVT session |  | 0.14 |  |  |
| VPVT_FAST_MEAN | Average of fastest 10% of reaction times during the vPVT session |  | 0.14 |  |  |
| VPVT_FAST_STD | Standard deviation of fastest 10% of reaction times during the vPVT session |  | 0.07 |  |  |
| VPVT_LAPSES | Count of reaction times > 500 ms | 1.00 | 0.16 | 0.35 |  |
| VPVT_M1 through VPVT_M10 | Average reaction time for each minute of the vPVT session |  | 0.09-0.17 |  |  |
| VPVT_L1 through VPT_L10 | Count of lapses for each minute of the vPVT session |  | 0.09-0.14 |  |  |
| VPVT_SLOPE | Slope of line fit through average of 1/RT per minute of vPVT session |  | -0.05 |  |  |
| VPVT_INTERCEPT | Intercept of line fit through average of 1/RT per minute of vPVT session |  | -0.14 |  |  |
| KSS | Karolinska sleepiness scale score from 1-9 |  | 0.04 | 0.38 | 0.01 |
| VAS_Alert | Level of alertness from 0 to 100 (0 = Sleepy, 100 = Alert) |  | -0.04 | -0.17 | -0.06 |
| VAS_Sad | Level of sadness from 0 to 100 (0 = Happy, 100 = Sad) |  | 0.04 |  | 0.08 |
| VAS_Calm | Level of calmness from 0 to 100 (0 = Excited, 100 = Calm) |  | 0.00 |  | -0.03 |
| ADD_Attempted | Number of attempted addition problems |  | -0.06 | -0.24 | -0.32 |
| ADD_PercentCorr | Percentage of correct addition problems |  | 0.01 |  | 0.03 |
| DSST_Attempted | Number of attempted digit-symbol substitutions |  | -0.08 | 0.44 | -0.46 |
| DSST_PercentCorr | Percentage of correct digit-symbol substitutions |  | 0.01 |  | 0.10 |
| DSST_MEANCORR | Mean reaction time of all correct responses on the DSST |  | 0.08 |  | 0.47 |
| DSST_MEANWRONG | Mean reaction time of all incorrect responses on the DSST |  | 0.04 |  | 0.04 |
| DSST_MEANOVER | Overall mean reaction time on the DSST |  | 0.08 |  | 0.47 |
| DSST_RATEOVER | Overall rate of response on the DSST |  | 0.08 |  | 0.47 |
| APVT_ANTICIPATIONS | Count of responses recorded prior to auditory stimulus presentation |  | -0.01 |  |  |
| APVT_ALL_MEAN | Average of all reaction times during the aPVT session |  | 0.18 | 0.38 |  |
| APVT_ALL_MED | Median of all reaction times during the aPVT session |  | 0.17 |  |  |
| APVT_ALL_STD | Standard deviation of all reaction times during the aPVT session |  | 0.12 |  |  |
| APVT_SLOW_MEAN | Average of slowest 10% of reaction times during the aPVT session |  | 0.15 |  |  |
| APVT_SLOW_STD | Standard deviation of slowest 10% of reaction times during the aPVT session |  | 0.08 |  |  |
| APVT_FAST_MEAN | Average of fastest 10% of reaction times during the aPVT session |  | 0.13 |  |  |
| APVT_FAST_STD | Standard deviation of fastest 10% of reaction times during the aPVT session |  | 0.07 |  |  |
| APVT_LAPSES | Count of reaction times > 500 ms |  | 0.15 | 0.37 |  |
| APVT_M1 through APVT_M10 | Average reaction time for each minute of the aPVT session |  | 0.11-0.16 |  |  |
| APVT_L1 through APVT_L10 | Count of lapses for each minute of the aPVT session |  | 0.06-0.13 |  |  |
| APVT_SLOPE | Slope of line fit through average of 1/RT per minute of aPVT session |  | -0.03 |  |  |
| APVT_INTERCEPT | Intercept of line fit through average of 1/RT per minute of aPVT session |  | -0.14 |  |  |

ADD, addition calculation test; aPVT, auditory psychomotor vigilance task; DSST, digit-symbol substitution task; KSS, Karolinska Sleepiness Scale; NA, not applicable; VAS, visual analog scale; vPVT, visual psychomotor vigilance task;
